# Cross-sectional study on the association between frailty and violence against community-dwelling elderly people in Brazil

**DOI:** 10.1590/1516-3180.2017.0203290817

**Published:** 2017-12-18

**Authors:** Mariane Santos Belisário, Flavia Aparecida Dias, Maycon Sousa Pegorari, Mariana Mapelli de Paiva, Pollyana Cristina dos Santos Ferreira, Fabrício Anibal Corradini, Darlene Mara dos Santos Tavares

**Affiliations:** I Undergraduate Student, Nursing Course, Universidade Federal do Triângulo Mineiro (UFTM), Uberaba (MG), Brazil.; II MSc. Doctoral Student, Postgraduate Course on Healthcare, Universidade Federal do Triângulo Mineiro (UFTM), Uberaba (MG), Brazil.; III MSc. Physiotherapist and Assistant Professor, Physiotherapy Course, Universidade Federal do Amapá (UNIFAP), Macapá (AP) Brazil.; IV MSc. Nurse and Assistant Professor, Technical Nursing Course, Instituto Federal de Educação Ciência e Tecnologia do Norte de Minas Gerais (IFNMG), Almenara (MG), Brazil.; V MSc. Doctoral Student, Postgraduate Course on Healthcare, Universidade Federal do Triângulo Mineiro (UFTM), Uberaba (MG), Brazil.; VI MD. Adjunct Professor, Department of Geography, Universidade Federal do Triângulo Mineiro (UFTM), Uberaba (MG), Brazil.; VII MD, PhD. Associate Professor, Undergraduate Nursing Program, Department of Nursing Education and Community Health, Universidade Federal do Triângulo Mineiro (UFTM), Uberaba (MG), Brazil.

**Keywords:** Elderly, Frail elderly, Violence, Geographic information systems, Spatial analysis

## Abstract

**BACKGROUND:**

The physical, emotional and cognitive limitations that may be present in the aging process, coupled with family unpreparedness, may lead to greater dependence among the elderly. This favors development of frailty syndrome and greater levels of violence against the elderly. The objective here was to analyze the association between violence against the elderly and frailty; and the geographic distribution of violence against the elderly according to the presence of frailty syndrome.

**DESIGN AND SETTING:**

Cross-sectional study on 705 community-dwelling elderly people in Uberaba (MG), Brazil.

**METHODS:**

The Fried frailty phenotype and conflict tactics scale were used. Data were analyzed using descriptive statistics, the chi-square test and a logistic regression model. The intensity of the events and the relationship between clusters of violence and frailty status were assessed by means of kernel estimation.

**RESULTS:**

The adjusted analysis indicated that pre-frailty and frailty were associated with physical and verbal aggression (odds ratio, OR = 1.51; 95% confidence interval, CI: 1.04-2.19; OR = 2.12; 95% CI: 1.29-3.47), frailty was associated with physical aggression (OR = 2.48; 95% CI: 1.25-4.94) and pre-frailty and frailty were associated with verbal aggression (OR = 1.48; 95% CI: 1.03-2.15; OR = 2.15; 95% CI: 1.31-3.52), respectively. Regardless of frailty status and its relationship with violence, clusters of occurrences were larger in similar regions in the southeastern part of the municipality; but superimposition of overlays relating to aggression showed that for frail individuals the clusters were smaller than for non-frail and pre-frail individuals.

**CONCLUSIONS:**

The condition of frailty was associated with greater chances of violence against the elderly.

## INTRODUCTION

Amid the expansion of longevity, propitiated through advances in medicine and improvements in living standards, elderly people have been experiencing new situations that are considered adverse, such as increasing numbers of cases of violence against them.^1^ Through the aging process, elderly people may become more susceptible to physical, emotional or cognitive limitations, which may lead to greater dependence.^2^ This, together with family unpreparedness, low socioeconomic status and histories of violence among relatives, may contribute towards occurrences of violence against them in this phase of life.^3^

The World Health Organization has adopted the definition of violence against the elderly proposed by the International Network for the Prevention of Elderly Abuse (INPEA). This definition states that such violence is “an act (single or repeated) or omission that causes harm or distress and which occurs in any relationship in which there is an expectation of confidence”.^4^

A multicenter study conducted among elderly people in different countries showed that 0.85% of the sample reported physical violence and 14.82% psychological violence. Female gender, low education levels, low income levels, multi-family living arrangements and lack of support from the partner, children and family were associated with cases of domestic violence, especially psychological violence.^5^

In Brazil, a review of the literature found that the prevalence of violence against the elderly ranged from 3.2% to 20.8%, depending on the cases reported and the region where each study was conducted. The types of event reported included psychological and physical violence, along with robbery. However, attention was drawn to the small number of studies conducted in this country on this subject, especially with regard to population-based studies, thus showing that there is a need to increase knowledge in this field.^6^

In this context, frailty among the elderly can be highlighted. This may lead to greater susceptibility to the risk of violence, which can be correlated with the level of functional dependence. This dependence increases the burden on caregivers who live in the same home, which may compromise the quality of care and interfere in family relationships.^7^

Another review of the literature^8^ identified the prevalence of frailty syndrome among the elderly, finding a range from 6.9% to 39.1%. This range of results was ascribed to differences between countries and between the types of places evaluated (in the community or in hospitals, healthcare units or long-stay institutions), along with the instruments used.^8^

Considering these data, it is important to identify cases of risk of violence against the elderly that are related to the presence of frailty syndrome. To do so, tools such as georeferencing constitute an important technological resource that can contribute towards ascertaining the dimensions of a particular problem, in terms of where people affected by the problem are living, within a defined geographical area. These data can contribute towards better organization of care services, through planning of healthcare actions and decision-making that are directed towards the target public.^9^

## OBJECTIVE

Based on the above, and considering the lack of studies evaluating maltreatment and/or violence against the elderly in the scientific literature, in relation to frailty syndrome, the objectives of the present study were to verify the association between violence against the elderly and frailty syndrome and to conduct a geographic analysis on violence against the elderly according to the presence of frailty syndrome.

## METHODS

### Study design, population and ethics

This was a household-based analytical and observational cross-sectional survey, developed among elderly residents in the urban area of the municipality of Uberaba (MG), Brazil. The sample size calculation used a prevalence of violence of 40%,^10^ an accuracy of 3.5% and a 95% confidence interval for a finite population of 36,703 elderly people, and the sample size thus determined was 738 individuals. A sampling loss of 20% was taken into consideration, and it was decided that the maximum number of interviews to be attempted would be 923.

To select the elderly subjects, a multistage cluster sampling technique was used. In the first stage, an arbitrary draw of 50% of the census tracts of the municipality of Uberaba was carried out by means of systematic sampling. The sample interval (SI) was calculated by using the following formula: SI = total number of census tracts/number of census tracts selected. In the second stage, the number of elderly people to be interviewed according to the sample calculation (n = 738), was divided by the number of census tracts in the municipality (204). This proportion was approximated to four elderly people per census tract, in order to obtain an approximately similar number within each census tract.

The inclusion criteria were that the subjects needed to: be aged 60 or older; live in the urban area; have no cognitive impairment; be able to walk, for which use of a walking aid was allowed (walking stick, crutch or Zimmer frame); and agree to participate in the study through signing a free and informed consent statement. The following were considered to be exclusion criteria and losses: failure to find the elderly individual, after three attempts by the interviewer; being hospitalized; neurological diseases that made evaluations impossible; failure to complete all the tests for frailty; and occurrence of census tracts without elderly people or in which the number of elderly people required was not reached. Thus, 705 elderly people were interviewed. Data were collected from January to April 2014, in the respective homes of these elderly individuals, on a single occasion, through a direct interview.

This study was approved by the Ethics Committee for Research on Human Beings of the Federal University of the Triângulo Mineiro through report no. 573.833. The interviews were conducted only after the interviewees had given their consent through signing a free and informed consent statement.

### Measurements

Initially, a cognitive assessment using the Mini-Mental State Examination (MMSE) was conducted. The cutoff point for cognitive decline took into consideration the respondents’ educational level and was taken to be 13 points if the subject was illiterate, 18 points or less if the subject had attended school for between one and 11 years and 26 points if the subject had had more than 11 years of education.^11^ An structured survey was used to characterize socioeconomic, clinical and health data. In addition, functional incapacity was measured using the Katz^12^ and Lawton-Brody^13^ questionnaires for basic activities of daily living (BADL) and instrumental activities of daily living (IADL), respectively.

Frailty syndrome was verified using the five items that were proposed by Fried et al.^14^ for describing the components of the frailty phenotype, as follows:

1. Unintentional weight loss, assessed by the question: “In the past year, did you unintentionally lose more than 4.5 kg (i.e. without diet or exercise)?”;
2. Diminished muscle strength, which was verified through handgrip strength using a manual hydraulic dynamometer (SAEHAN model). Three measurements expressed in kilogram-force (kgf) were obtained, with one-minute intervals between them. The mean value of the three measurements was used and the cutoff points proposed by Fried et al. were used;^14^
3. Self-reported exhaustion and/or fatigue: measured by means of two questions on the Brazilian version of the Center for Epidemiological Studies depression scale (CES-D): item 7 (“I felt everything I did was an effort”) and item 20 (“I could not get going”). The elderly subjects who scored two or three in either of these questions fulfilled the frailty criteria for this item;^15^
4. Walking slowness, in which the time (in seconds) that was taken to walk a distance of 4.6 m was assessed. Three measurements expressed in seconds were made, and the mean value was used. The cutoff points used were as proposed by Fried et al.;^14^
5. Low physical activity level, as assessed using the International Physical Activity Questionnaire (long version), with adaptation for elderly people.^16^ The classification used for this component was that elderly people who spent 150 minutes or more per week doing physical activities were considered active, while those who spent zero to 149 minutes per week doing physical activity were considered inactive.

Elderly subjects with three or more of the above items were classified as frail; those with one or two items were classified as pre-frail; and those for whom all the tests were negative were considered to be robust or non-frail.^14^

Violence was assessed using the translated and validated Brazilian version of the conflict tactics scale.^17^ This instrument is composed of 19 questions encompassing negotiation, psychological aggression, physical assault, sexual coercion and injury. The score range is from 0 to 19. Psychological or physical violence was considered to have occurred when the elderly subjects reported that they had been the victims of at least one item on the verbal aggression subscale (questions 4-9) and physical aggression subscale (questions 10-19).^17^

The variables studied were: socioeconomic data; number of diseases; number of medications; BADL and IADL dependency; frailty syndrome; and physical and verbal aggression.

### Statistical analysis

The data were analyzed through the Statistical Package for the Social Sciences (SPSS) software, version 17.0. To characterize the population, the statistical analysis was performed using the absolute and percentage frequency distribution for the categorical variables.

To ascertain associations between violence and socioeconomic, clinical and health variables, the chi-square and Student t tests were used. Logistic regression was used to examine the condition of frailty that was associated with physical and psychological violence, with adjustments for age, sex, number of diseases and number of medications. The significance level (α) was set at 5%.

For the spatial analysis, the MapInfo Professional software, version 9.5, and the TerraView software, version 3.3.1, were used. A georeferenced database was built to spatialize the data, by using the Geographic Information System (GIS) tools through the ArcGis 10.2 application. The intensities of the events and the relationships of clusters of violence with frailty status (number of events per unit area) were assessed through kernel estimates, with an adaptive algorithm for the radius of the quartic function. The maps generated for each event were subjected to a reclassification process, followed by multicriterion analysis with weighted overlays, with the aim of ascertaining whether the different events overlapped and what their common occurrence area was. It should be noted that all products generated were adjusted to the same horizontal datum (SIRGAS 2000) and that the Universal Transverse Mercator (UTM) coordinate system was used.

## RESULTS

Among the interviewees (n = 705), the highest percentages were female (66.8%), in the age group 60├70 years (43.1%) were married (42%), had a monthly income of one minimum monthly wage (45%), had had 4├8 years of schooling (36.5%) and lived with a companion (78.9%). Regarding violence, 20.9% of the elderly subjects reported that they had suffered verbal aggression, 7.9% physical aggression and 21.13% physical and/or verbal aggression. Regarding their condition of frailty, it was found that 15.9% (n = 112) were frail, 52.2% (n = 368) were pre-frail and 31.9% (n = 225) were non-frail.

Associations between the following variables and types of violence were observed: individual monthly income of one minimum wage (physical aggression); living with another person and IADL dependency (physical and/or verbal aggression and verbal aggression); and a large number of diseases (physical and/or verbal aggression, physical aggression and verbal aggression). Regardless of the type of aggression, the highest proportion of the victims comprised pre-frail elderly people. However, the proportion of frail elderly people who reported having suffered physical and/or verbal aggression, physical aggression and verbal aggression was higher than the proportion of frail elderly people who reported that they had not suffered any aggression, as shown in Table 1.

**Table 1:** Socioeconomic, clinical and health variables according to distribution of types of violence. Uberaba (MG), Brazil, 2014

Variables	Physical and/or verbal aggression		Physical aggression		Verbal aggression	
	Yes	No	Yes	No	Yes	No
	n (%)	n (%)	n (%)	n (%)	n (%)	n (%)
Sex						
Male	44 (29.5)	190 (34.2)	19 (33.9)	215 (33.1)	43 (29.1)	191 (34.3)
Female	105 (70.5)	366 (65.8)	37 (66.1)	434 (66.9)	105 (70.9)	366 (65.7)
P*	0.285		0.903		0.229	
Age range (years)						
60├70	70 (47.0)	243 (42.1)	29 (51.8)	275 (42.4)	69 (46.6)	235 (42.2)
70├80	53 (35.6)	204 (36.7)	19 (33.9)	238 (36.7)	53 (35.8)	204 (36.6)
80 or over	26 (17.4)	118 (21.2)	8 (14.3)	136 (21.0)	26 (17.6)	118 (21.2)
P*	0.470		0.318		0.521	
Length of schooling (years)						
Illiterates	25 (16.8)	110 (19.8)	8 (14.3)	127 (19.6)	25 (16.9)	110 (19.7)
1├4	37 (24.8)	129 (23.2)	16 (28.6)	150 (23.1)	37 (25.0)	129 (23.2)
4├8	58 (38.9)	199 (35.8)	26 (46.4)	231 (35.6)	57 (38.5)	200 (35.9)
8	14 (9.4)	35 (6.3)	3 (5.4)	46 (7.1)	14 (9.5)	35 (6.3)
9 or more	15 (10.1)	83 (14.9)	3 (5.4)	95 (14.6)	15 (10.1)	83 (14.9)
P*	0.328		0.166		0.343	
Marital Status						
With a companion	12 (8.1)	41 (7.4)	4 (7.1)	49 (7.6)	12 (8.1)	41 (7.4)
Without a companion	137 (91.9)	515 (92.6)	52 (92.9)	600 (92.4)	136 (91.9)	516 (92.6)
P*	0.780		0.585		0.759	
Monthly income (in minimum wages)						
No income	10 (6.7)	53 (9.5)	3 (5.4)	60 (9.2)	10 (6.8)	53 (9.5)
< 1	7 (4.7)	9 (1.6)	6 (10.7)	10 (1.5)	7 (4.7)	9 (1.6)
1	71 (47.7)	246 (44.2)	27 (48.2)	290 (44.7)	70 (47.3)	247 (44.3)
1├3	49 (32.9)	182 (32.7)	20 (35.7)	211 (32.5)	49 (33.1)	182 (32.7)
3├5	8 (5.4)	41 (7.4)	0 (0)	49 (7.6)	8 (5.4)	41 (7.4)
> 5	4 (2.7)	25 (4.5)	0 (0)	29 (4.5)	4 (2.7)	25 (4.5)
P*	0.163		< 0.001		0.169	
Living arrangements						
Alone	20 (13.4)	129 (23.2)	8 (14.3)	141 (21.7)	20 (13.5)	129 (23.2)
Not alone	129 (86.6)	427 (76.8)	48 (85.7)	508 (78.3)	128 (86.5)	428 (76.8)
P*	0.009		0.191		0.011	
Number of diseases (mean ± SD)	6.62 ± 3.79	5.47 ± 3.36	7.39 ± 3.92	5.57 ± 3.41	6.61 ± 3.8	5.48 ± 3.36
P^†^	< 0.001		< 0.001		0.001	
Number of medications (mean ± SD)	3.53 ± 0.520	3.36 ± 2.75	3.57 ± 2.89	3.38 ± 2.76	3.51 ± 2.88	3.37 ± 2.75
P^†^	0.520		0.631		0.576	
Dependency in BADL (mean ± SD)	0.03 ± 0.21	0.02 ± 0.15	0.03 ± 0.18	0.02 ± 0.16	0.03 ± 0.21	0.02 ± 0.15
P^†^	0.448		0.597		0.439	
Dependency in IADL (mean ± SD)	1.05 ± 1.44	0.72 ± 1.25	03.91 ± 1.10	0.77 ± 1.32	1.04 ± 1.45	0.72 ± 1.25
P^†^	0.012		0.461		0.013	
Frailty						
Non-frail	45 (30.2)	180 (32.4)	14 (25.0)	211 (32.5)	44 (29.7)	181 (32.5)
Pre-frail	67 (45.0)	301 (54.1)	25 (44.6)	343 (52.9)	67 (45.3)	301 (54.0)
Frail	37 (24.8)	75 (13.5)	17 (30.4)	95 (14.6)	37 (25.0)	75 (13.5)
P*	0.003		0.008		0.003	

P < 0.05; *chi-square test; †Student t test; BADL = basic activities of daily living; IADL = instrumental activities of daily living; SD = standard deviation.

The adjusted analysis indicated that the conditions of pre-frailty and frailty were both associated with higher odds ratios for physical and/or verbal aggression, while the condition of frailty alone was associated with physical aggression and the conditions of pre-frailty and frailty were both associated with verbal aggression (Table 2).

**Figure 1:**
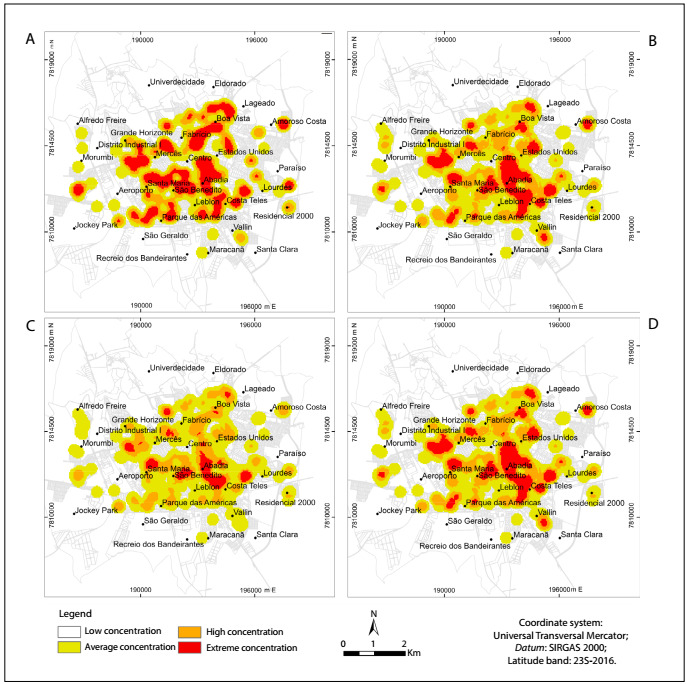
A. Map of where non-frail elderly people were living; 1B. With overlay regarding verbal aggression; 1C. With overlay regarding physical aggression; 1D. With overlay regarding verbal/physical aggression. Uberaba (MG), Brazil, 2014. Concentration data relate to qualitative information.

The geospatial analysis showed that the non-frail elderly people were present in several regions of the municipality (Figure 1A). Superimposition of overlays relating to verbal aggression (Figure 1B) and physical aggression (Figure 1C) showed that non-frail elderly people suffering from these problems were concentrated in the southeastern region of the municipality, but that non-frail elderly people suffering from physical aggression presented at lower density than those suffering from verbal aggression. The overlay showing physical/verbal aggression (Figure 1D) in relation to areas with non-frail elderly people demonstrated that the individuals suffering from physical/verbal aggression were concentrated in the southeastern region of the municipality.

**Table 2:** Logistic regression model according to the classification of violence according to frailty levels. Uberaba (MG), Brazil, 2014

Frailty levels	Physical and/or verbal aggression			Physical aggression			Verbal aggression		
	OR	95% CI	P	OR	95% CI	P	OR	95% CI	P
Non-frail	1			1			1		
Pre-frail									
Non-adjusted	1.44	1.01-2.08	0.047	1.39	0.80-2.41	0.240	1.42	0.99-2.05	0.058
Adjusted	1.51	1.04-2.19	0.029	1.45	0.83-2.55	0.191	1.48	1.03-2.15	0.036
Frail									
Non-adjusted	2.12	1.36-3.30	0.001	2.54	1.38-4.67	0.003	2.14	1.37-3.34	0.001
Adjusted	2.12	1.29-3.47	0.003	2.48	1.25-4.94	0.010	2.15	1.31-3.52	0.002

OR = odds ratio; 95% CI = 95% confidence interval; P < 0.05; 1 = reference category; adjustments were made for age, sex, number of diseases and number of medications.

**Figure 2:**
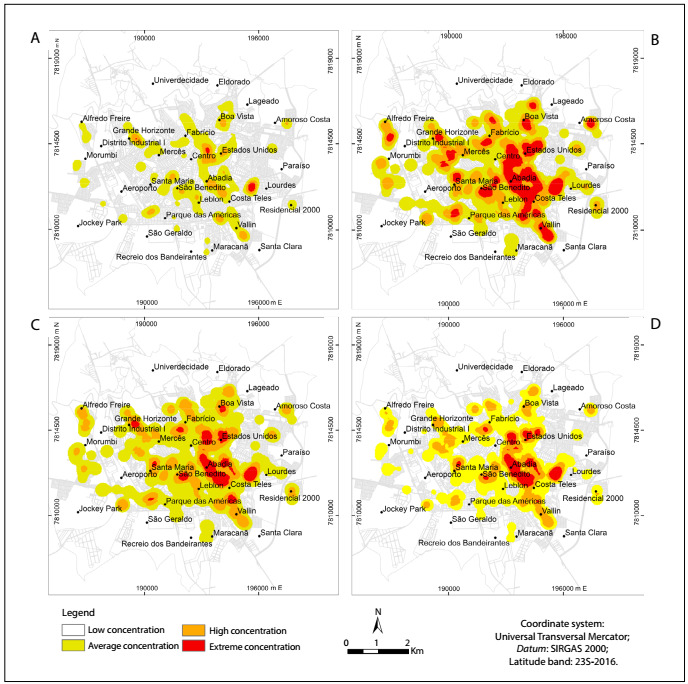
A. Map of where pre-frail elderly people were living; 2B. With overlay regarding verbal aggression; 2C. With overlay regarding physical aggression; 2D. With overlay regarding verbal/physical aggression. Uberaba (MG), Brazil, 2014. Concentration data relate to qualitative information.

Pre-frail elderly people were concentrated in the southeastern region and close to the central region (Figure 2A). Superimposition of overlays relating to verbal aggression (Figure 2B) and physical aggression (Figure 2C) showed that the region with pre-frail elderly people suffering from these problems was the southeastern region. The areas shown by the overlay relating to physical/verbal aggression coincided with the southeastern region, but were smaller (Figure 2D**)**.

**Figure 3:**
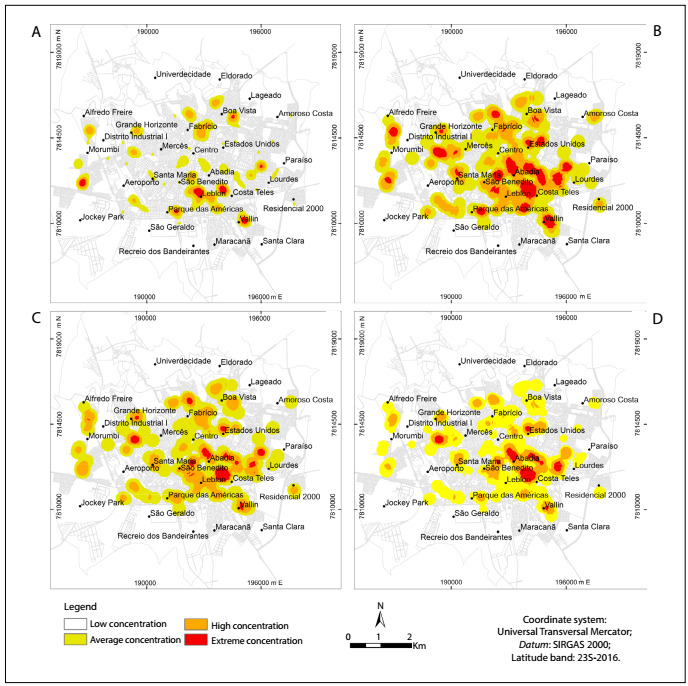
A. Map of where frail elderly people were living; 1B. With overlay regarding verbal aggression; 1C. With overlay regarding physical aggression; 1D. With overlay regarding verbal/physical aggression. Uberaba (MG), Brazil, 2014. Concentration data relate to qualitative information.

Frail elderly people were concentrated mainly in the southern region of the municipality (Figure 3A). Superimposition of overlays relating to verbal aggression (Figure 3B) and physical aggression (Figure 3C) showed that the southeastern region presented the largest concentration of frail elderly people. Overlaying of physical/verbal aggression on areas with frail elderly people showed that these areas coincided with the southeastern region, but were smaller than the area in which individuals suffered from verbal aggression (Figure 3D).

## DISCUSSION

This study demonstrated that conditions of frailty were associated with higher odds ratios for physical and/or verbal aggression, physical aggression and verbal aggression. Regardless of frailty status and its relationship with violence, clusters of occurrences were larger in the southeastern part of the municipality; but for frail individuals the clusters were lower than for non-frail and pre-frail individuals.

In relation to the sociodemographic data, the present study showed that the elderly people investigated here followed the trend observed in other studies. i.e. the subjects of the present study were predominantly female and younger elderly people who were married, had had little schooling and were living with other people.^5^^,^^18^

Regarding the types of violence, a Brazilian study^19^ and a multicenter study^5^ showed different prevalences of physical and psychological violence. This may have been related to the definitions for violence that were used, along with social differences.^20^

A survey conducted in the city of Campinas, state of São Paulo, Brazil,^21^ showed lower prevalence than the present study, such that 9.1% of the community-dwelling elderly people were frail, while the percentage of pre-frail elderly people was similar (51.8%). However, a systematic review with meta-analysis revealed higher prevalence of frail conditions (19.1%) in countries in the Latin American and Caribbean region.^22^

The results regarding socioeconomic data and occurrence of violence were consistent with those from other studies, for the variables of sex,^19^ age group,^23^ marital status,^24^ schooling^19^^,^^23^ and income.^5^ The sociodemographic context that was identified suggests that the issue of violence remains linked to the most vulnerable groups, i.e. women and individuals of lower social and economic levels. With regard to age group, younger adults are assumed to have a lower degree of dependence because their younger age makes it easier to seek help and to identify more cases of violence.^25^ On the other hand, in relation to marital status, although this was not investigated in the present study, married women might present some dependence on their spouses, which might increase the possibility of becoming victims of their partners.^25^

The results relating to the proportion of frail elderly people among the elderly people who reported occurrences of aggression, and the association between the condition of frailty and occurrences of physical and/or verbal aggression, physical aggression and verbal aggression, are corroborated by the findings from a systematic review that showed that worse health conditions or frailty were risk factors for abuse among community-dwelling elderly people.^26^

Other studies have mentioned the expression “frailty”, although without any established conceptual and operational definition, and have correlated this with neglect of care and signs of abandonment among hospitalized elderly people;^27^ or with specific components of vulnerability to abuse among elderly people, with regard to disability and mortality rates.^28^ One point that needs to be mentioned is that the Brazilian Ministry of Health’s recommended definition for frail elderly people or for individuals in a frail condition includes scenarios of living with situations of domestic violence, among other situations envisaged.^29^

Thus, identification of conditions of frailty and care for frail elderly people should include directed and expanded investigation of whether cases and/or situations of violence might exist. In addition, screening and/or early diagnosis may favor implementation of preventive measures aimed at addressing frailty syndrome and occurrences of violence against elderly people.

In the spatial analysis, regardless of frailty status and its relationship with violence, the clusters of elderly were largest in the same region of the southeast of the municipality. This suggests that other factors, possibly relating to space, may have interfered with these variables. It should be noted that the areas highlighted present concentrations of people with low incomes and have lower percentages of literate people than elsewhere.^30^

Less schooling and low income characterized the elderly victims of violence.^31^ Individuals with higher levels of education are likely to suffer less violence, since they have a minimum critical level that enables them to reduce or avoid abuse.^32^ In addition, groups with higher education seek other sectors, such as medical and legal services, to solve the problem.^33^ Thus, during professional care, it is necessary to consider the level of schooling of elderly patients, in order to facilitate understanding of the guidelines for the rights of elderly people.

In a bibliographical survey on the social determinants of violence, in relation to the health of vulnerable populations in Latin America, it was found that, among other factors, excess urban violence is related to income inequality.^34^ Thus, in view of the complexity of violence, there is a need for a broader view that considers its interdependence among individual, relational and cultural determinants.^35^

It is noteworthy that we were unable to find any other spatial analysis studies correlating conditions of frailty with violence among elderly people, which thus limits the possibilities for discussing this. On the other hand, we found some surveys on violence against women, and these showed that there was greatest occurrence in geographical spaces with less favored social conditions or in situations of greater social inequality.^36^^,^^37^

Hence, mapping of the areas at greater risk can direct the attention of the public authorities towards actions focusing on geographical spaces identified through this process.^36^ Identification of places where there are concentrations of elderly people in conditions of frailty/pre-frailty who suffer violence can guide healthcare provision at the primary care level.

There is a need to prepare professionals at this level of care to meet this demand. Professional and institutional supervision services need to be offered by public managers, in order to improve the team’s ability to listen to patients’ complaints and encourage greater professional participation in making decisions and connecting services together.^35^

In this regard, it should be pointed out that there need to be links between the judicial, safety, health, social and educational support systems, along with community awareness campaigns focusing on the different types of violence that the elderly are exposed to.^31^

The limitations of the present study included its cross-sectional design, in which the data were collected at a single time. This made it impossible to ascertain the causal relationship between the variables. Nonetheless, despite the characteristics of the methodology used, the results from this study add knowledge to this topic. Variables that have been little explored in the scientific literature, namely frailty and violence against the elderly were analyzed here. Thus, the findings from the present study add support for proposals for preventive interventions and behaviors directed towards addressing the conditions of violence against the elderly and frailty among the elderly.

## CONCLUSION

The proportion of frail elderly people who reported occurrences of aggression was higher than the proportion of them who did not report such occurrences. The condition of frailty was associated with higher odds ratios for physical and/or verbal aggression, physical aggression and verbal aggression. Regardless of frailty status and its relationship with violence, the clusters of elderly people were larger in similar regions of the southeastern part of the municipality.
